# Prenatal genesis of layer II doublecortin expressing neurons in neonatal and young adult guinea pig cerebral cortex

**DOI:** 10.3389/fnana.2015.00109

**Published:** 2015-08-10

**Authors:** Yan Yang, Mi-Xin Xie, Jian-Ming Li, Xia Hu, Peter R. Patrylo, Xue-Gang Luo, Yan Cai, Zhiyuan Li, Xiao-Xin Yan

**Affiliations:** ^1^Department of Anatomy and Neurobiology, Central South University School of Basic MedicineChangsha, China; ^2^Department of Nursing in Internal Medicine, Xiangtan Vocational and Technical CollegeXiangtan, China; ^3^Neuroscience Research Center, Changsha Medical UniversityChangsha, China; ^4^Center for Integrated Research in Cognitive and Neural Sciences, Southern Illinois University School of MedicineCarbondale, IL, USA

**Keywords:** BrdU birth-dating, cerebral evolution, doublecortin, neurogenesis, neuroplasticity

## Abstract

Cells expressing doublecortin (DCX+) occur at cortical layer II, predominantly over the paleocortex in mice/rats, but also across the neocortex among larger mammals. Here, we explored the time of origin of these cells in neonatal and 2-month-old guinea pigs following prenatal BrdU pulse-chasing. In the neocortex, BrdU+ cells birth-dated at embryonic day 21 (E21), E28, and E35 laminated over the cortical plate with an inside-out order. In the piriform cortex, cells generated at E21 and E28 occurred with a greater density in layer II than III. Many cells were generated at later time points until birth, occurring in the cortex without a laminar preference. DCX+ cells in the neocortex and piriform cortex partially co-colocalized with BrdU (up to 7.5%) in the newborns after pulse-chasing from E21 to E49 and in the 2 month-old animals after pulse-chasing from E28 to E60/61, with higher rates seen among the E21-E35 groups. Together, layer II DCX+ cells in neonatal and young adult guinea pigs may be produced over a wide prenatal time window, but mainly during the early phases of corticogenesis. Our data also show an earlier establishment of the basic lamination in the piriform relative to neocortical areas in guinea pigs.

## Introduction

Cells with antigenic, morphological, and electrophysiological properties of immature neurons are present at layer II (with some in upper III) in developing and adult mammalian cerebrum (Bonfanti and Nacher, 2012). Initially these cells were described in the paleocortex of adult rats with immunohistochemistry for polysialic acid neural cell adhesion molecule (PSA-NCAM) (Seki and Arai, 1991). Later this same population of cells was reported in the piriform cortex with immunolabeling of doublecortin (DCX) in rats (Nacher et al., 2001) and other antigen markers in non-human primates (Bernier et al., 2002). During the past several years, these cells have been discovered in the paleocortex and neocortex in an increasing list of mammals including mouse (Zhang et al., 2010; Klempin et al., 2011); guinea pig (Xiong et al., 2008; Luzzati et al., 2009; He et al., 2014), rabbit (Luzzati et al., 2009), cat (Cai et al., 2009; Varea et al., 2011), dog (De Nevi et al., 2013), nonhuman primates (Cai et al., 2009; Bloch et al., 2011; Marlatt et al., 2011) and human (Liu et al., 2008; Cai et al., 2009; Srikandarajah et al., 2009; Fung et al., 2011), as well as some less commonly studied species such as lesser hedgehog tenrecs (Alpár et al., 2010) and members of superorder Afrotheria (Patzke et al., 2014). In nonhuman primates, layer II DCX expressing (DCX+) cells persist into advanced ages in some associative cortical areas (Zhang et al., 2009). Together, these data point to a certain conserved neurobiological role for layer II immature neurons in mammalian cerebrum (Decimo et al., 2012).

Many puzzling facets concerning layer II DCX+ neurons are yet to be reconciled. The time by which these cells are generated differs considerably as shown among recent studies. They are found to be largely born prenatally in adult rats (Gómez-Climent et al., 2008), while it is also suggested that they can be produced in adult life in rodents and other mammals (Bernier et al., 2002; Pekcec et al., 2006; Liu et al., 2008; Guo et al., 2010; Xiong et al., 2010). The site of origin of these cells is suggested to be either local (Guo et al., 2010; Xiong et al., 2010) or distant, e.g., the subventricular zone (SVZ) (Bernier et al., 2002; Shapiro et al., 2007). The fate of these cells has been considered to be principal neurons (Luzzati et al., 2009; Guo et al., 2010) or interneurons (Xiong et al., 2008; Cai et al., 2009; Zhang et al., 2009; Klempin et al., 2011). Moreover, the developmental trajectory of these cells appears to be “exceptionally” long in light of our current understanding of neuronal development or adult neurogenesis (Bonfanti and Nacher, 2012).

Given the species-related difference of layer II DCX+ cells between the paleo- and neo-cortices, it is important to explore the formation of these cells in context of cortical development/evolution. Corticogenesis involves an inside-out lamination of the cortical plate, as established in the neocortex of various mammals, including mouse (Angevine and Sidman, 1961), rat (Bayer et al., 1991; Ignacio et al., 1995), cat (Luskin and Shatz, 1985), ferret (Jackson et al., 1989) and nonhuman primates (Rakic, 1974). An early [3H]-thymidine autoradiographic study in rat has shown that the primary olfactory (piriform) cortex is also laminated with an “inside-out” pattern (Bayer, 1986). Less is clear about cell genesis relative to lamination in the paleocortex in comparison with neocortex in most, especially phylogenetically advanced, mammals.

As a tailless rodent, guinea pigs show some neuroanatomical features often typically seen in higher mammals (e.g., carnivorous and primates), rather than in small rodents such as mice and rats, including a presence of the lateral sulcus, a relatively large encephalization quotient and an occurrence of layer II DCX+ immature neurons in the neocortex (Rice et al., 1985; Herculano-Houzel, 2007; Xiong et al., 2008). Guinea pigs are born following a fairly long pregnancy (about 9 weeks), in consideration of their body size or lifespan, relative to other mammals. Thus, they may serve as a convenient and cost-affordable model for studying mammalian cortical morphogenesis and neuronal development. In the present study we used guinea pigs to explore the time of origin of layer II DCX+ cells in the neo- and paleo-cortices of neonatal and 2-month-old young adult offspring, following 5′ bromodeoxyuridine (BrdU) pulse-chasing at weekly intervals from embryonic day 21 (E21) to just before birth.

## Materials and methods

### Animals, BrdU injection and tissue processing

Experimental procedures were approved by the Ethics Committee of Central South University Xiangya School of Medicine for animal care and use. Animals, prenatal BrdU administration and tissue processing were described in detail in a recent report (Liu et al., 2015). Briefly, two doses (50 mg/kg/dose, 12 h apart, i.p.) of 5-bromodeoxyuridine (BrdU) (B5002, Sigma-Aldrich, St Louis, MO, USA) were given to time-pregnant mothers (*n* = 3/point) at embryonic day 14 (E14), E21, E28, E35, E42, E49, E56, and E60/61 for the expected offspring. Brains of the postnatal guinea pigs were examined at the day of birth, defined as postnatal day 0 (P0), and at P60 (*n* = 3–4/point). Brains were coded according to the embryonic day receiving BrdU injection and the day of brain perfusion (e.g., E21-P0). The mothers that received BrdU injections at E14 for the expected offspring suffered aborted pregnancy (with no postnatal brains available for study).

Animals were perfused under deep anesthesia (sodium pentobarbital, 100 mg/kg, i.p.) via the ascending aorta with 4% paraformaldehyde in 0.01 M phosphate-buffered saline (pH 7.4, PBS). Brains were removed, postfixed in the perfusion fixative overnight, and immersed in 30% sucrose until tissue sank for cryoprotection. The forebrains were cut frontally at 30 μm thickness in a cryostat, with 24 sets of sections collected in order in cell culture plates. Sets of sections were processed for Nissl stain as well as DCX, BrdU and DCX/BrdU immunolabeling, respectively.

### Immunohistochemistry and immunofluorescence

DCX and BrdU immunolabeling was carried out using the avidin-biotin-peroxidase (ABC) method. Briefly, sections were bleached in 1% H_2_O_2_ in PBS containing 5% normal sera (horse serum for DCX; rabbit serum for BrdU) and 0.3% Triton X-100 for 45 min, with sections subjected to BrdU labeling additionally treated in 1 × SSC and 50% formamide at 65°C for 1 h and in 2N HCl at 37°C for 30 min. Subsequently, sections were reacted overnight at 4°C with the goat anti-DCX (Santa Cruz Biotech., CA, USA, sc-8066, diluted at 1:2000) or the rat anti-BrdU (AbD Serotec, Raleigh, NC, USA, MCA2060, 1:2000) antibody, respectively. After further reaction with biotinylated secondary antibodies (1:400) and ABC reagents (1:400) (Vector Laboratories, Burlingame, CA, USA), immunoreaction product was visualized in 0.003% H_2_O_2_ and 0.05% diaminobenzidine. In each experiment, sections passing the mid-septum and mid-hippocampus were included in the staining process except for the exposure to the primary antibodies (incubated with the antibody dilution buffer instead). These sections were used to obtain the cut-off levels of nonspecific immunolabeling in the follow-up densitometric analysis.

For double immunofluorescence, sections were first incubated free-floating in PBS containing 5% donkey serum and 0.1% Triton X-100 for 1 h at room temperature, followed by an overnight reaction with the DCX and BrdU antibodies at 4°C. The immunoreactivity was detected following an incubation with a pair of Alexa-Fluor® or DyLight™ 488 conjugated donkey anti-goat and Alexa-Fluor® or DyLight™ 594 conjugated donkey anti-rat IgGs (1:200, Jackson ImmunoRes. Lab., Inc., West Grove, PA, USA). The immunolabeled sections were counterstained with bisbenzimide (Hoechst 33342, 1:50000, Sigma-Aldrich, St. Louis, MO), washed and mounted with anti-fading medium before microscopic examination.

### Imaging, cell count and data processing

Sections from the entire rostrocaudal dimension of the cerebrum were examined on an Olympus fluorescent BX53 microscope equipped with a digital imaging system (CellSens Standard, Olympus, Japan) and a Nikon confocal fluorescent microscope (Nikon, DIGITAL ECLIPSE C1 plus). Sections at the levels of mid-septum to mid-hippocampus were used for quantitative imaging analyses. BrdU labeling over the somatosensory neocortex and piriform cortex was imaged with the same exposure setting using the 10 × objective, followed by a reconstruction of each area by image montage. Optic density (o.d.), expressed as digital light unit per square millimeter (DLU/mm^2^), was measured over the cortex (to be defined further in the result section with illustration) using the OptiQuant system (Parkard Instruments, Meriden, CT, USA). As such, the o.d. values reported from the immunolabeled sections represented the “total density” of the labeled profiles, whereas the values from the sections processed in the absence of the primary antibody were defined as “non-specific density.” The o.d. of “specific labeling” in a given area of interest was thus calculated by subtracting the “non-specific” from “total” measured densities. To quantify the rate of BrdU colocalization among DCX+ cells, confocal images collecting fluorescence in ~5 μm tissue depth (1.7 μm × 3 scans) were captured at 20 × (covering 450 × 335 mm^2^ actual tissue area) using two sections per brain. While moving along the pial side of the cortex, images were taken for every one in three (i.e., #1, #4, #7….) neighboring microscopic fields, resulting in approximately 16 images per hemisphere containing more than 1000 DCX+ cells. The numbers of DCX+ cells and those colabeled with BrdU were counted in each image, with the sums and colocalization rate calculated accordingly. Data from the neocortex and piriform cortex of individual brains were presented with dot graphs.

### Statistical analysis and figure preparation

Means of specific optic density or percentage values of colocalization were input into the Prism spreadsheet (Prism GraphPad 4.1, San Diego, CA, USA) for graph preparation. The means and standard derivations (S.D.) were calculated for individual groups, with statistical analysis performed using the Kruskal-Wallis test. Minimal significant level of difference was set at *p* < 0.05. Graphic and image figures were assembled with Photoshop 7.1.

## Results

### Laminar distribution of BrdU labeled cells in P0 and P60 guinea pig cerebrum

**Figure 1:**
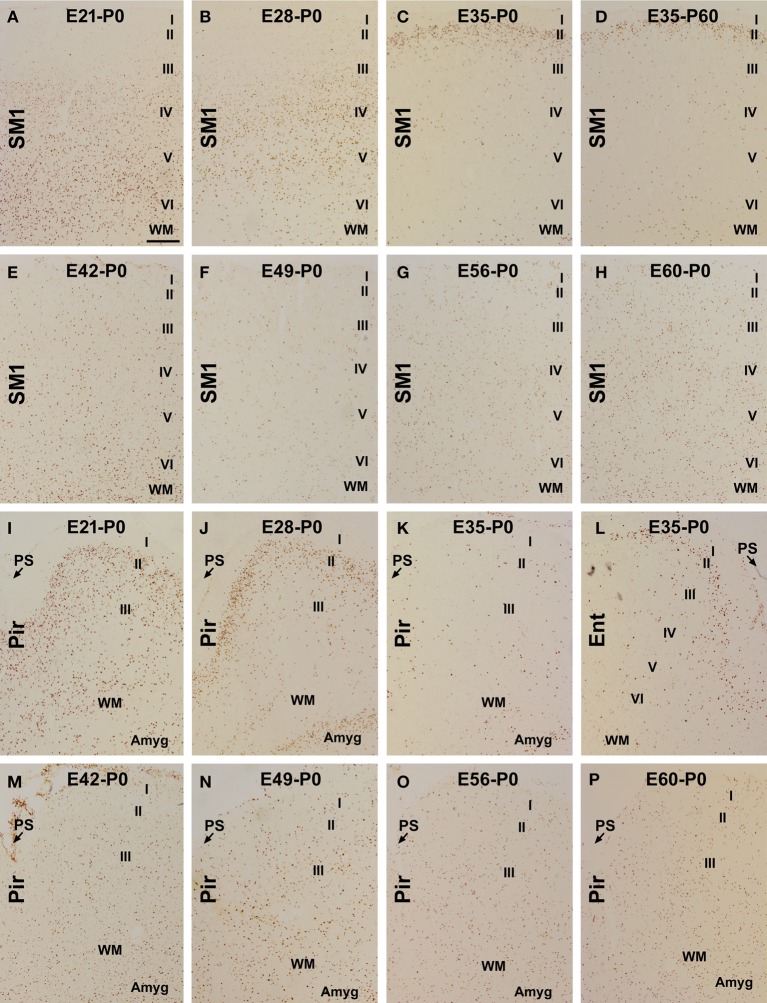
**Laminar distribution of BrdU labeled (BrdU+) cells in postnatal guinea pig cerebral cortex birth-dated at various embryonic time points**. Shown are representative images of BrdU+ cells in the primary somatosensory (SM1) and piriform cortex (Pir) from animal groups coded according to the embryonic day (**E**, e.g., E21, E28…E60/61) receiving BrdU dosing and the day of brain perfusion, with the day of birth defined as postnatal day 0 (P0). BrdU+ cells are largely located in the infragranular **(A)**, middle **(B)** and superficial **(C,D)** layers of the cortical plate of the neocortex in the E21-P0, E28-P0, E35-P0, and E35-P60 groups, respectively. Among other groups, the labeled cells are present across the cortical layers without a preferential lamination **(E–H)**. In the piriform cortex, BrdU+ cells occur more densely in layer II than III in the E21-P0 **(I)** and E28-P0 **(J)** groups, with a much reduced density in the E35-P0 group **(K)**. In the E35-P0 as well as the remaining groups, BrdU+ cells also lack a differential laminar distribution in the piriform cortex **(K,M–P)**. Note the reduced cell density in layer II in the entorhinal cortex (Ent) relative to SM1 in the E35-P0 group **(C,L)**. Additional abbreviation: I–VI, cortical layers; WM, white matter; PS, piriform sulcus. Amyg: amygdala. Scale bar = 100 μm in **(A)** applying to other panels.

**Figure 2:**
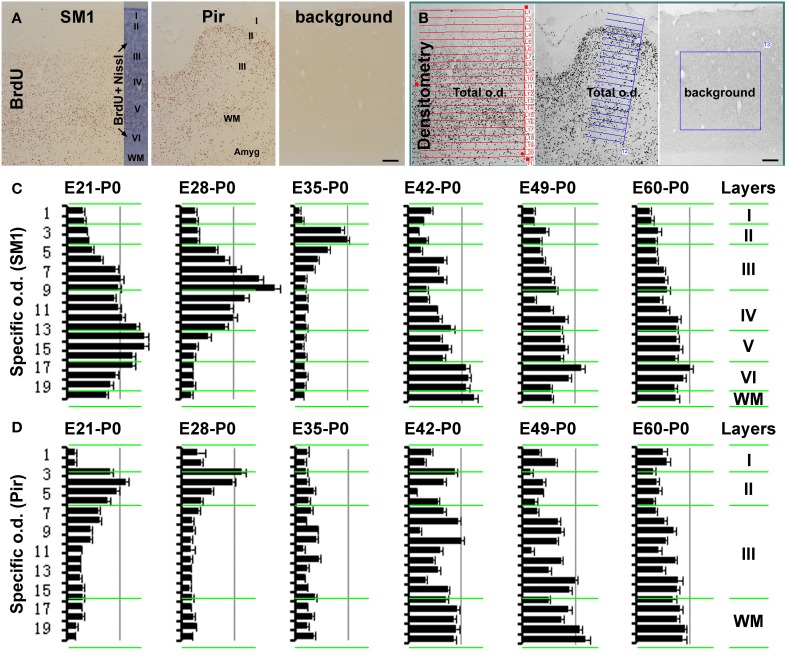
**Histograms showing the relative laminar densities of BrdU labeled cells in the primary somatosensory (SM1) and piriform (Pir) cortices in the neonatal groups received prenatal BrdU dosing (as defined in Figure 1)**. **(A)** (immunolabeling image) and **(B)** (screen-print image) show the densitometric method used to obtain specific optic densities (o.d.) in the SM1 and Pir using a section passing the mid-hippocampus in each animal. Total optic densities are obtained over 20 cortical tangential sectors, adjusted to extend from the pia to the white matter (WM), while a background density is obtained from a batch-processed section in the absence of the primary antibody, with specific optic density calculated by subtracting the later from the former values. The density in the piriform cortex is obtained in the middle 1/3 part of this region, by rotating the measuring template with its long axis perpendicular to the pial surface. Bar graphs **(C,D)** represent the mean specific optic densities of the cells in individual tangential sectors for different animal groups (*n* = 3/group). The approximate cortical laminar boundaries (marked on the left of the bar graphs) relative to the tangential sectors are assessed in an immunolabeled sections with Nissl counterstain **(A)**. Highest density occurs in the deep, middle, and superficial layers of the cortical plate in the neocortex in the E21-P0, E28-P0, and E35-P0 groups, respectively **(C)**. In the piriform cortex, layer II shows a higher density than III in the E21-P0 and E28-P0 groups **(C)**. The specific density appears non-differential over the cortical layers in the SM1 and Pir among other groups **(C,D)**. Scale bar = 100 μm in **(A,B)**.

We examined BrdU labeled (BrdU+) cells (nuclei) across the rostrocaudal and dorsomedial to ventrolateral dimensions of the cerebrum in P0 and P60 guinea pigs received prenatal BrdU injections. Overall, the laminar distribution of BrdU+ cells showed more or less differences between animal groups with BrdU pulse-chasing at different prenatal time points, in either the neonatal (e.g., E21-P0 vs. E28-P0) or 2-month-old (e.g., E21-P60 vs. E28-P60) groups. However, the laminar pattern was similar for the neonatal and 2-month-old groups that received BrdU injections at the same embryonic time (e.g., E35-P0 vs. E35-P60, as shown in Figures 1C,D for an example). Therefore, we chose to present the laminar distribution data from the neonatal groups mainly, for simplicity (Figures 1, 2). It should be also noted that for brains of the same time point group, the laminar distribution pattern of BrdU+ cells was largely comparable from the rostral to caudal levels for the neocortical, entorhinal (transitional), and piriform (paleocortex) regions, individually, whereas, differences in laminar distribution of the labeled cells existed between these phylogenically different cortical regions. The cortical region and chasing-time related differences in cell distribution were well represented in sections from the mid-septal to mid-hippocampal levels. Accordingly, we assessed BrdU labeled cells using these sections, with a lamina-based densitometry in the primary somatosensory (SM1) and piriform (Pir) cortical areas, representing the neo- and paleo-cortical regions, respectively (Figures 2A,B).

In the neocortex, BrdU+ cells were predominantly localized to the infragranular layers in the E21-P0 group (Figures 1A, 2A–C), layers III/IV in the E28-P0 group (Figures 1B, 2C) and layer II in the E35-P0 group (also in the E35-P60 group, Figures 1C, 2C). Unlike the above groups, BrdU labeled cells seen in the neocortex of animals pulse-chased at E42, E49, E56, and E60/E61 did not exhibit a preferential laminar distribution pattern in the neonatal offspring (Figures 1E–H, 2C).

In the piriform cortex, BrdU+ cells in the E21-P0 group occurred in layers II and III, denser in the former (Figures 1I, 2A,B,D). Similarly, the cells were more densely distributed in layer II relative to III in the E28-P0 group (Figures 1J, 2D). In contrast, the labeled cells were dramatically reduced in number but lost the pattern of preferential lamination in the E35-P0 group (Figures 1K, 2D). As with the neocortex, BrdU+ cells in the piriform cortex of animals pulse-chased at E42, E49, E56, and E60/E61 did not exhibit a preferential laminar distribution pattern (Figures 1M–P, 2D). It should be noted that BrdU+ cells in the entorhinal cortex exhibited a similar laminar distribution pattern relative to the neocortex among the groups in general. However, the amount of cells in layer II was noticeably reduced relative to the neocortex in the E35-P0 group (Figures 1C,L). Thus, a transitional pattern in overall density of BrdU+ cells in layer II was seen when comparing the parietotemporal to the entorhinal, and to the piriform cortices (Figures 1C,K,L).

### Overall cortical morphology and distribution of DCX+ cells in neonatal guinea pigs

**Figure 3:**
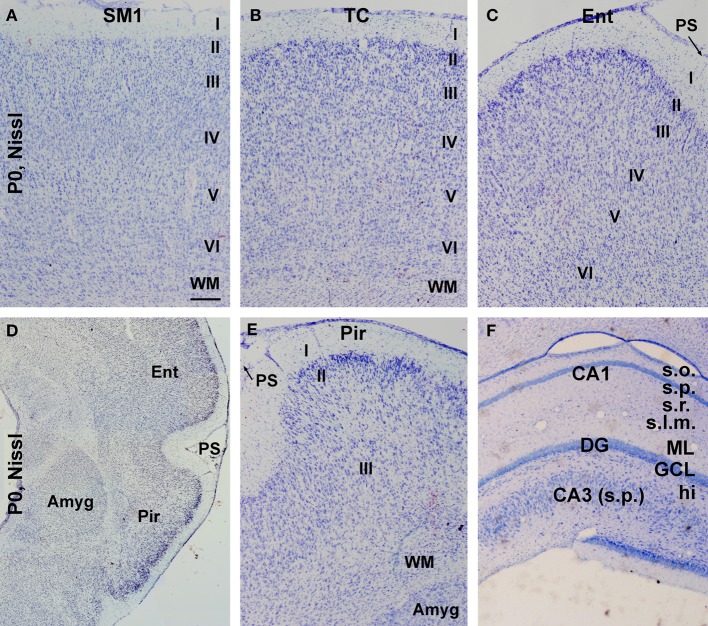
**Nissl stain images showing the overall laminar architecture in cerebral subregions in neonatal guinea pigs**. **(A–C)** show that the primary somatosensory (SM1), temporal (TC), and entorhinal (Ent) cortices are organized as six cellular layers. **(D)** is a low magnification image showing the amygdala (Amyg) and surrounding entorhinal and piriform cortices, with the latter containing three cellular layers **(E)**. An adult-like cellular lamination is seen in the hippocampal CA1 and CA3 sectors as well as the dentate gyrus **(F)**. Additional abbreviation: I–VI, cortical layers; WM, white matter; PS, piriform sulcus; s.o., stratum oriens; s.p., stratum pyramidale; s.r., stratum radiatum; s.l.m., stratum lacunosum-moleculare; ML, molecular layer; GCL, granule cell layer; hi, hilus. Scale bar = 100 μm in **(A)** applying to **(B,C,E,F)**; equivalent to 500 μm for **(D)**.

The overall morphological maturity of cerebral structures in the neonatal guinea pigs was assessed in Nissl stain preparations. Similar, to that seen in the adult animals (http://www.brainmuseum.org/specimens/rodentia/guineapig/index.html), the neocortex of the newborns could be divided into six cellular layers, while the piriform cortex was arranged as layers I-III, and the entorhinal cortex showed a transition of lamination between the parietotemporal and piriform cortical regions (Figures 3A–E). The amygdalar complex with its subdivisions was identifiable medially to the entorhinal and piriform cortices (Figure 3D). The laminar/cellular architecture of the hippocampus and dentate gyrus also showed an apparently adult-like pattern (Figure 3F).

**Figure 4:**
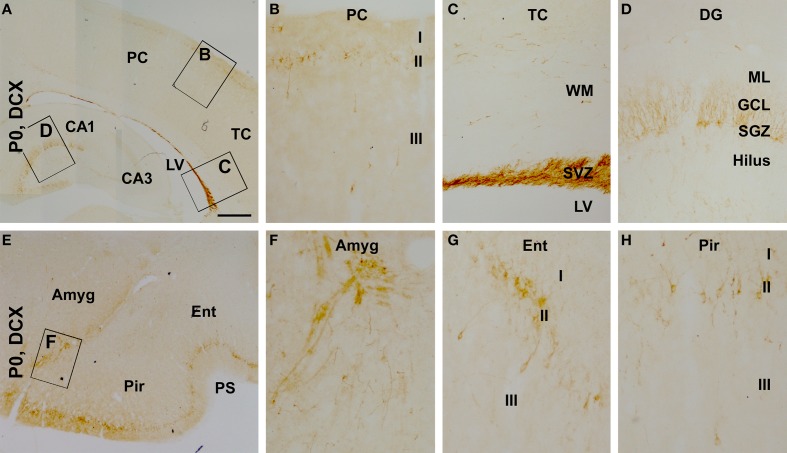
**Distribution of doublecortin-expressing (DCX+) profiles in neonatal (postnatal day 0, P0) guinea pig cerebral subregions**. DCX+ cells are visible at low magnification in the cortex, the subventricular zone (SVZ) and dentate gyrus (DG) **(A)**. At higher magnifications, DCX+ cells in the cortex align along layer II, with a few seen in layer III **(B)**. The SVZ next to the lateral ventricle (LV) contains densely packed DCX+ cells, with some cells also seen in the white matter (WM) **(C)**. DCX+ cells in the DG are densely packed along the subgranular zone (SGZ), with their dendrites extending across the granule cell layer (GCL) into the molecular layer (ML) **(D)**. DCX+ cells are also found in the amygdala (Amyg) **(E,F)**. DCX+ cells in the piriform and entorhinal cortices also occur largely in layer II **(G,H)**, with a higher density relative to the neocortex **(B)**. Other abbreviations are as defined in Figure 3. Scale bar = 500 μm in **(A)** applying to **(E)**, equivalent to 100 μm for **(B–D,F,G)**.

We also assessed the regional/laminar distribution of DCX+ cells in several cerebral regions in the newborns by referring to existing adult data. Layer II DCX+ cells in the neonatal guinea pig cerebral cortex exhibited a regional pattern that was largely comparable to that seen in juvenile and adult animals (Xiong et al., 2008), with a dorsal to ventral, low-to-high, gradient in the overall amount of labeling from the parietotemporal to the entorhinal, and to the piriform cortical areas (Figures 4A–H). DCX+ cells were densely packed along the subventricular zone, with some located in the white matter (Figures 4A,C). A large amount of DCX+ cells was present at the subgranular zone (SGZ) of the dentate gyrus with their dendritic processes extending across the granule cell layer (GCL) toward the molecular layer (ML) (Figures 4A,D). In addition, as described in primates (Bernier et al., 2002; Zhang et al., 2009; Martí-Mengual et al., 2013), DCX+ cells occurred in the amygdala, especially along its border to the lateral ventricle (Figures 4E,F).

### BrdU colocalization in layer II DCX+ cells in P0 and P60 guinea pig cerebrum

**Figure 5:**
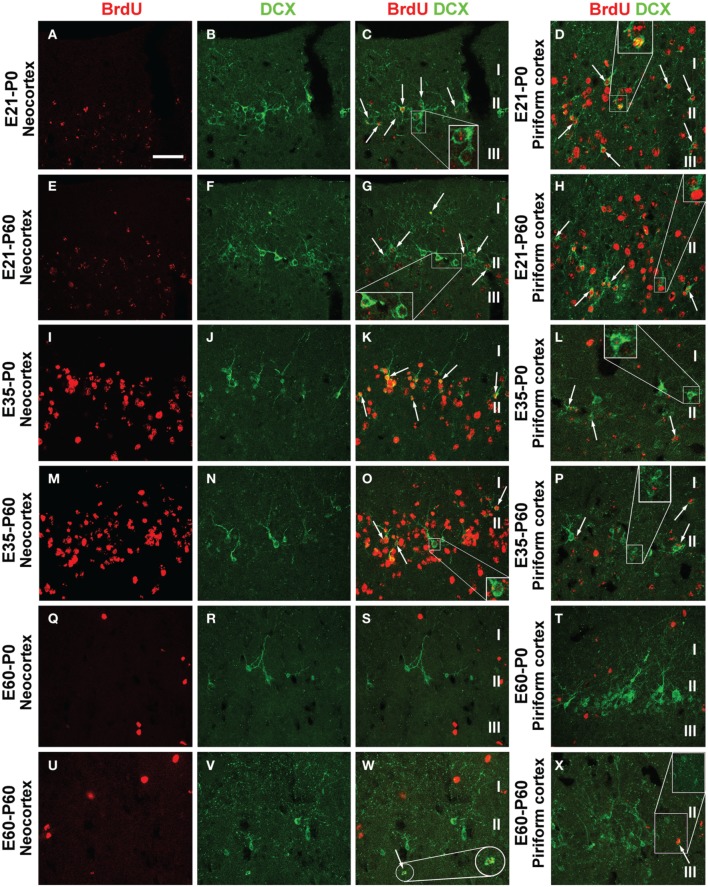
**Confocal immunofluorescent images showing colocalization of BrdU in layer II doublecortin expressing (DCX+) cells in the neocortex and piriform cortex in representative time groups as indicated**. Examples of double-labeled cells are indicated by arrows and enlarged inserts in the panels **(A–X)** as labeled. BrdU immunoreactivity may be densely packed or appear as granules in the nucleus of single and double-labeled cells. The morphology and labeling intensity of DCX+ cells also show a great extent of variability. Scale bar = 50 μm in **(A)** applying for panels **(B–X)**.

In examination of double immunofluorescent sections we observed a partial BrdU colocalization among layer II DCX+ cells across the cortical hemisphere in most groups of the P0 and P60 offspring (Figure 5, showing images from representative groups). In a given brain, double-labeled cells could be detected in the parietotemporal, entorhinal, and piriform cortical areas. Consistent with previous observations (Xiong et al., 2008; Cai et al., 2009), layer II DCX+ cells exhibited a great extent of variability in morphology and labeling intensity. Among the BrdU+ cells including those coexpressing DCX, BrdU labeling in the nucleus appeared densely packed, or granular and punctate (Figures 5C,D,G,H,K,L,O,P; enlarged inserts).

**Figure 6:**
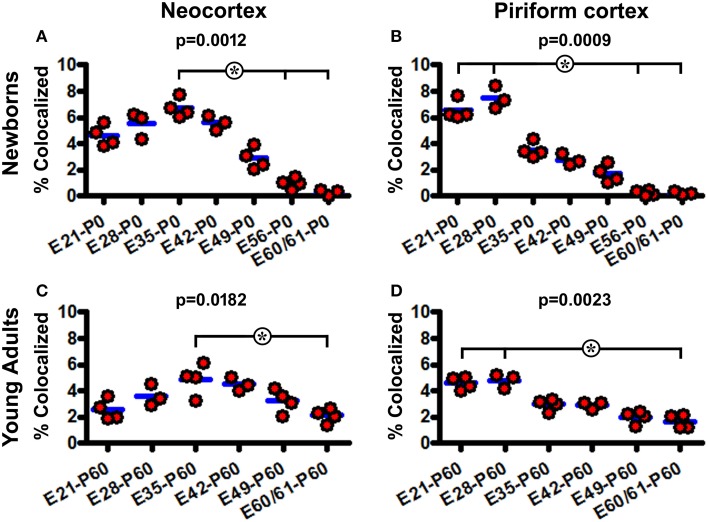
**Quantification of BrdU colocalization in layer II DCX+ cells in neonatal and young adult guinea pigs following prenatal pulse-chasing**. Dot graphs **(A–D)** summarize the colocalization rates (% of BrdU+/DCX+ relative to total DCX+ cells) in the neocortex **(A,C)** and piriform cortex **(B,D)** in the newborns **(A,B)** and 2-month-old **(C,D)** offspring. Each dot represents the colocalization rate calculated for one animal, with the blue bar indicating the group mean. The *p*-values are obtained by Kruskal-Wallis test, showing statistically significant overall difference of medians in the groups, with *post-hoc* test results (^*^p < 0.05) between subgroups marked.

The extent of BrdU colocalization among layer II DCX+ cells was quantified in the neocortex collectively over the areas dorsomedial to the piriform sulcus, and in the piriform cortex. In the P0 groups, the rates of BrdU colabeling in the DCX+ cell population in the neocortex were estimated to be 4.6 ± 0.81%, 5.5 ± 0.99%, 6.8 ± 0.74%, 5.6 ± 0.54%, 2.9 ± 0.81%, 0.9 ± 0.41%, and 0.3 ± 0.30% following BrdU pulse-chasing at E21, E28, E35, E42, E49, E56, E60/61, respectively. Kruskal-Wallis test indicated an overall significant difference of the medians (*P* = 0.0012, Gaussian approximation; K-W statistic = 21.98), with *post-hoc* (Dunn's multiple comparison) test showed a difference for the E35 relative to E56 and E60/61 groups (Figure 6A). The colocalization rates in the piriform cortex were 6.5 ± 0.74%, 7.5 ± 0.84%, 3.5 ± 0.60%, 2.8 ± 0.43%, 1.7 ± 0.67%, 0.2 ± 0.18%, and 0.2 ± 0.12% following BrdU pulse-chasing at E21, E28, E35, E42, E49, E56, E60/61, respectively. There was also an overall difference in the medians (*P* = 0.0009; K-W statistic = 22.68), with a difference for the E21 and E28 relative to E56 and E60/61 groups by *post-hoc* test (Figure 6B). Among the P60 groups, the colocalization rates in the neocortex were 2.6 ± 0.75%, 3.6 ± 0.81%, 4.9 ± 1.19%, 4.5 ± 0.52%, 3.2 ± 0.92%, and 2.1 ± 0.53% for the E21, E28, E35, E42, E49, and E60/61 groups, respectively, with an overall difference of the medians (*P* = 0.0182; K-W statistic = 13.62) and a difference between the E35 and E60/61 groups by *post-hoc* test (Figure 6C). In the piriform cortex, the colocalization rates were 4.6 ± 0.49%, 4.8 ± 0.56%, 2.9 ± 0.45%, 2.9 ± 0.27%, 2.0 ± 0.50%, and 1.7 ± 0.50% for the E21, E28, E35, E42, E49, and E60/61 groups, respectively (*P* = 0.0023; K-W statistic = 18.63, with *p* < 0.05 for the E21 and E28 vs. E60/61 groups by *post-hoc* test) (Figure 6D).

## Discussion

Cortical morphogenesis and neuronal development are important research issues for understanding of cerebral functions and dysfunctions. The cortical plate is laminated inside-out in mammalian neocortex, likely relating largely to the production and migration of principal neurons. Precursors of principal neurons derive from the subventricular germinal zones, migrate radially and settle down in cortical layers VI to II sequentially (Angevine and Sidman, 1961; Molyneaux et al., 2007; Rakic, 2009). The development of cortical interneurons may involve more complex processes (Chu and Anderson, 2015). During early cortical development, GABAergic precursors from subpallial structures enter the cortical primordium and migrate tangentially (and likely then radially) to reach their final laminar positions (Lavdas et al., 1999; Ang et al., 2003; Tanaka et al., 2006; Wonders and Anderson, 2006; Petanjek et al., 2008; Gelman et al., 2011). Early-born interneurons include those developing into the intrinsic GABAergic subgroups in layer I and the subcortical white matter (Bayer and Altman, 1990; Yan et al., 1992, 1996; Cao et al., 1996; Zecevic and Rakic, 2001; Liu et al., 2015). Notably, GABAergic neurons may be born at late stages of cortical development in small rodents (Soriano et al., 1992; Hevner et al., 2004; Rymar and Sadikot, 2007; Inta et al., 2008; Riccio et al., 2012) as well as other species including human (Hladnik et al., 2014; Arshad et al., 2015; Liu et al., 2015). The present study adds new data in light of the above general/current understanding of mammalian cortical morphogenesis and neuronal formation.

### Prenatal cell genesis relative to lamination in guinea pig cerebral cortex

While corticogenesis has been studied in many mammals by [3H]-thymidine birth-dating and other methods (e.g., Angevine and Sidman, 1961; ALTMAN, 1963; Rakic, 1974; Luskin and Shatz, 1985; Jackson et al., 1989; Bayer et al., 1991; Ignacio et al., 1995), comparative data on cell genesis relative to lamination between different cortical domains remain limited to date. In rats (21 day pregnancy), cells to build the basic layers of the piriform cortex are produced at the same or slightly earlier embryonic days relative to the neocortex, with earlier-born cells settled in layer III and later-born cells in layer II (Bayer, 1986). A recent BrdU birth-dating study in mice indicates that a larger amount of layer II cells in the piriform cortex are born at E12 than E14 and E16 (Sarma et al., 2011).

The present study reveals that cells birth-dated at E21, E28 and E35 reside orderly in deep to superficial layers of the cortical plate in the neonatal and young adult guinea pig neocortex, consistent with an inside-out lamination pattern. However, in the piriform cortex cells generated at both E21 and E28 reside more densely in layer II than III, while cells birth-dated at E35 are greatly reduced over these layers. These findings suggest that the basic lamination is completed earlier in the piriform than neocortical regions in guinea pigs (by at least 1 week). To our knowledge, this is the first characterization of such a fairly differential timeline of cell genesis relative to lamination between the neo− and paleo-cortex in mammals.

The present study also shows a considerable occurrence of BrdU+ cells in neonatal and 2 month-old guinea pig cortex following pulse-chasing around (piriform cortex) and after (neocortex) the 5th embryonic week. These later-born BrdU+ cells do not show a preferential laminar or regional distribution. While some of the late-born cells would contribute to a subpopulation of the layer II DCX+ cells (this study) and type II nicotinamide adenine dinucleotide phosphate diaphorase positive neurons in postnatal guinea pigs (Liu et al., 2015), much work is needed to illustrate the full spectrum of the fate(s) of these cells. With much of the process of cell proliferation, migration and differentiation taken place prenatally, guinea pig cerebral cortex appears considerably mature-looking, showing typical laminar/cellular organization as seen in the adult. In particular, an adult-like distribution pattern of DCX+ cells in the cortex, amygdala, and hippocampal formation is readily established by birth (Xiong et al., 2008).

### Prenatal genesis of cortical layer II DCX+ cells in neonatal and young adult guinea pigs

A previous study indicates that PSA-NCAM+ cells in adult rat (assessed at 3 month of age) piriform cortex are largely produced between E13.5 and E15.5 (Gómez-Climent et al., 2008). In the present study we observe a partial colocalization (up to 7.5%) of BrdU among layer II DCX+ cells in neonatal and 2-month old guinea pigs following pulse-chasing at multiple prenatal time points, with higher colocalization rates seen in the E21-E35 groups. As discussed above, in guinea pigs cells destined to layer II are predominantly birth-dated at E21 and E28 in the piriform cortex and at E35 across the neocortex. Thus, layer II DCX+ neurons in neonatal and young adult guinea pigs appear to be mainly generated during early corticogenesis, i.e., before and by the time the final outward migratory waves of cells to the cortical plate are produced. Since double-labeled cells are detectable in 2-month old offspring with BrdU pulse-chasing as early as E21, layer II DCX+ cells appear to represent a unique population of cortical cells that may differentiate with a fairly slow pace.

BrdU/DCX colocalization is rare in the neonatal groups received BrdU dosing at E56 and E60/61. This might imply a developmental time-delay of DCX expression following BrdU incorporation. It is worth noting that some double-labeled cells, especially among those birth-dated at the earlier embryonic time points (E21-E35), show a granular or punctate BrdU labeling in the nucleus. This pattern might suggest that BrdU was incorporated into the cells during a partial period of DNA replication. Alternatively, BrdU signal may be diluted due to multiple cell divisions or during the course of postmitotic neuronal differentiation. In fact, it becomes difficult to track BrdU labeling in prenatally-chased offspring surviving to 4–6 months of age (own experience).

In summary, the present study shows an earlier completion of the basic lamination in the paleo- than neo-cortical regions in guinea pigs, with some cortical cells also being generated at late gestational stages. While layer II DCX+ cells in the neo− and paleo-cortices of neonatal and young adult animals may be produced over a wide prenatal time period, they appear to be largely formed during the early phases of cortical development.

### Conflict of interest statement

The authors declare that the research was conducted in the absence of any commercial or financial relationships that could be construed as a potential conflict of interest.
